# Psychological Distress among College Students: Role of Food Insecurity and Other Social Determinants of Mental Health

**DOI:** 10.3390/ijerph17114118

**Published:** 2020-06-09

**Authors:** Monideepa B. Becerra, Benjamin J. Becerra

**Affiliations:** 1 Department of Health Science and Human Ecology, Center for Health Equity, California State University, San Bernardino, CA 92407, USA; 2 Department of Information & Decision Sciences, California State University, San Bernardino, CA 92407, USA; bbecerra@csusb.edu

**Keywords:** psychological distress, university students, food insecurity, mental health

## Abstract

Food insecurity is a major social determinant of health and an assessment of how it may impact college students’ mental health is imperative, as well as differential associations by self-identified gender. A cross-sectional survey was used among college students of a mid-size minority-serving institution with a final sample size of 302 participants aged 18 years or above. Descriptive, bivariate, and multivariable regressions were conducted, by gender, to assess the role of food insecurity (United States Department of Agriculture (USDA) six-item questionnaire), on mental health outcomes (Kessler-6 scale and self-perception). All the statistical analyses were conducted in SPSS version 24 (IBM, Corp.; Armonk, NY, USA) with an alpha less than 0.05 used to denote significance. Among those with food insecurity, the odds of reporting psychological distress (odds ratio (OR) = 3.645, *p* < 0.05) and an average to very poor self-perceived mental health status (OR = 2.687, *p* <0.05) were higher compared to their food-secure counterparts, with the results consistent in a gender-specific analysis as well. Compared to men, however, women had higher odds of psychological distress (OR = 2.280, *p* < 0.05), as well as reporting average to very poor self-perceived mental health statuses (OR = 2.700, *p* < 0.05). Among women, any alcohol use in the past 12 months (OR = 2.505, *p* < 0.05) and a low self-perceived physical health status (OR = 3.601, *p* < 0.05) were associated with an average to very poor self-perceived mental health status. Among men, a low perceived physical health status was associated with higher odds of psychological distress (OR = 3.477, *p* < 0.05). The results of our study highlight that food insecurity should be considered a social determinant of mental health wellbeing. In addition, gender-specific trends in mental health highlight the need for targeted interventions for prevention and treatment.

## 1. Background

The World Health Organization (WHO) defines mental health as “a state of well-being in which the individual realizes his or her own abilities, can cope with the normal stresses of life, can work productively and fruitfully, and is able to make a contribution to his or her community” [1]. This demonstrates a paradigm shift in understanding health, from not just absence of illness but to an overall sense of wellbeing. Despite such efforts to make a comprehensive definition, along with global efforts to improve mental health status, one in four adults are impacted by a mental illness or neurological disorder at some point in their life [2]. Furthermore, in the U.S. one in five adults live with a mental illness [3]. According to the Centers for Disease Control and Prevention (CDC), nearly four percent of adults in the U.S. had serious psychological distress in the past 30 days. In addition, 56.8 million primary diagnoses related to mental, behavioral, and neurodevelopmental disorders were reported in 2016 [4].

One major population that has been shown to be disproportionately impacted by mental illness are young adults. According to the National Institute of Mental Health [4], the prevalence of any mental illness, which is defined as “a mental, behavioral, or emotional disorder,” was substantially higher among those aged 18–25 years (25.8%) when compared to adults aged 26–49 years (22.2%) or 50 years or older (13.8%). Similarly, the prevalence of serious mental illness, defined as “a mental, behavioral, or emotional disorder resulting in serious functional impairment, which substantially interferes with or limits one or more major life activities”, was also higher among those aged 18–25 years (7.5%) as compared to those aged 26–49 years (5.6%) or 50 years or older (2.7%). On the other hand, young adults were less likely to receive mental health services for any or serious mental illness when compared to other age groups. For example, only 38.4% of 18–25-year-olds with any mental illness received services compared to 43.3% of those aged 26–49 years and 44.2% of those aged 50 years or more [3]. This is further supported by empirical studies that have shown a high rate of mental illness among young adults, especially college students. For example, using the College Student Mental Health Survey, Soet and Sevig [5] demonstrated that 30% of their sample reported seeking counseling for mental health. Likewise, in comparing college-aged participants to non-college-aged counterparts, results from the 2001–2002 National Epidemiologic Survey [6] on Alcohol and Related Conditions showed that, while the college-aged group had lower rates of alcohol/drug/tobacco use, they were also less likely to receive treatment, bringing to attention the potential role of access. Undoubtedly, mental illness is a public health issue among young adults and understanding the factors that can lead to such negative mental health outcomes is critical.

In recent years, food insecurity, defined by the United States Department of Agriculture (USDA) as “household-level economic and social condition of limited or uncertain access to adequate food” [7], has been highlighted as a key social determinant of health [8]. For example, food-insecure adults with chronic illnesses are known to have poorer healthcare outcomes, including increased hospital visits, medication underuse, and prescription delays [9,10,11,12]. Likewise, food insecurity has been associated with negative health outcomes, including obesity and dietary patterns [13,14,15,16,17]. For example, Leung et al. noted that food-insecure adults were more likely to have a higher consumption of high-fat dairy products, salty snacks, meat, and sugar-sweetened beverages [18], while Nguyen et al. noted that food security was related to a higher Healthy Eating Index [19]. Despite such evidence on the negative impact of food insecurity, the understanding of how food insecurity relates to the mental health of young adults, especially college students, remains limited.

The current empirical evidence notes that food insecurity is becoming a growing public health issue among young adults, especially college students. For example, among 557 undergraduate students in the Southeastern U.S., Gaines et al. noted a 14% prevalence of food insecurity [20]. Likewise, in a study among University of California undergraduate and graduate studies, Martinez et al. noted the prevalence of food insecurity to be 42% [21]. The authors further noted various risk factors for food insecurity, including age, racial/ethnic background, childhood experiences of food insecurity, as well as receiving financial aid. Such food-insecure college students also had higher rates of negative outcomes, including lower academic performance and difficulty with concentration, among others [21]. Previous data from a minority-serving institution further noted the food insecurity prevalence to be nearly 32%, with almost 20% of college students reporting that they skipped or reduced meal size and 18% noting that food did not last long enough, while nearly 10% went hungry [22]. Additionally, among 237 undergraduate students surveyed in a public mid-Atlantic large university, Payne-Sturges et al. noted that 15% of the participants were food-insecure, while another 16% were at risk of becoming food-insecure. Similar to previous literature, the authors of this study also noted specific risk factors to being food-insecure, including belonging to a minority racial/ethnic group and receiving financial aid, in addition to housing insecurity [23]. Undoubtedly, food insecurity is a major social determinant of health, and an assessment of how it may impact college students’ mental health is imperative. Finally, much of the literature on food insecurity does not address gender-specific trends, despite current empirical evidence noting the gender-specific prevalence of mental illness, including among college students [24,25]. Likewise, the literature has shown gender differences in food insecurity, with a higher health burden among females [26].

(1) Objective: the objective of this study was to assess the role of food insecurity, in addition to additional social determinants, on the mental health outcomes of college students by self-identified gender.
(2) Hypothesis: based on the previous literature, we hypothesize that food-insecure college students will likely have more negative mental health outcomes with a higher burden noted for females.

## 2. Methods

### 2.1. Participants

Participants were selected from general education courses in a variety of departments across campus to ensure the diversity of the study population. General education (GE) classes were selected based on the class size (at least 20 students or more), variety of majors that took the course (at least two different majors were required to take the GE course), and the willingness of instructors to provide a scope of data collection. All the enrolled students were given extra credit for participation, and consent forms were obtained prior to the start of the survey. To remain compliant with the Institutional Review Board (IRB)-approval protocol, we did not link any names or identifiable information to the answers. We were approved for a maximum number of participants (350), and as such we left the survey open until we reached the sample size. Our study participants were recruited from a mid-sized, four-year, public institution of higher education. The majority of the population were first-generation Hispanic and commuters to the institution. In our study, we focused on adult college students, with no age cap. This was primarily because much of the young adult literature focuses on the inclusion of minors [27,28], and thus does not adequately reflect college students. Given increasing trends in adult learners, we felt that the inclusion of all adult (18 years or older) college students would help highlight the particular trends in this population, independent of the definition of young adult. No exclusion criteria beyond aged 18 or above were established, and thus all the students enrolled in the selected classes were eligible to participate. A total of six participants were excluded because their consent either could not be deciphered; there was confusion about which age category they selected; or they were in a gender minority group with a low sample size, which could result in accidental identification. As such, a total of 302 participants were included in this study.

### 2.2. Instrument

A quantitative close-ended survey was developed with validated measures. The outcome variable of interest in this study was mental health status, defined as self-perceived mental health status and psychological distress measured using Kessler-6 scale, a validated survey instrument to assess mental illness [29]. In this study, the psychological distress level was first categorized as no psychological distress, mild to moderate psychological distress, and serious psychological distress (SPD). A score of 13 or higher was considered as SPD, while 8–12 was considered mild to moderate psychological distress [30]. Given that the focus of this study was to evaluate the prevalence of psychological distress and factors associated with such an outcome, the presented results focused on yes versus no in the presence of psychological distress as the outcome of interest. In addition, the self-perceived mental health status was assessed as excellent, good, average, poor, and very poor. Given the distribution of responses, it was categorized as excellent/good versus average to very poor. These self-reported variables were consistent with previous literature on college students [22] and with the California Health Interview Survey [31] on general status questions as well.

The primary independent variable of interest in this study was food insecurity, based on USDA’s six-item questionnaire [32], which was categorized as food-secure versus food-insecure. Additionally, the following independent variables were included: self-identified gender (man or woman, with other groups removed from the study analysis due to the low sample size and potential to identify the respondents), age (18–20 years, 21–23 years, 24 or more years), ethnicity (Hispanic, non-Hispanic), employment status (working full time, working part-time, not working), country of birth (foreign born, U.S. born), any alcohol use in the past 12 months (yes, no), and self-perceived physical health status (excellent/good, average/poor/very poor). If a sample size was less than five, we did not include it in our study or collapsed it with another group to remain compliant with our ethical compliance guidelines. For example, less than five identified as other gender, and thus were excluded from the study. Age groups were categorized based on the natural breakpoints in the distribution. Given that the majority identified as Hispanic, the rest were combined into the category non-Hispanic. Given that our institution was primarily Hispanic-serving, we excluded a race-only data analysis, though we acknowledge the limitation of such an analysis.

### 2.3. Procedure

The selected GE courses were given out the survey in hard-copy format. The first page of the survey included a consent form, which was approved by our IRB. Students who wanted an alternative extra credit assignment were also given the alternate option instead of the survey. All the data were collected in a closed box and transferred directly to the principal investigator’s office and stored in a locked cabinet, per IRB approval. Each data file was extracted, and the data was manually entered into Microsoft Excel and then transferred to SPSS version 24 (IBM Corp.; Armonk, NY, USA). This study was approved by the Institutional Review Board of the California State University, San Bernardino.

### 2.4. Data Analysis

The population characteristics were evaluated using descriptive statistics. The association between mental health outcomes and each independent variable was further assessed using a chi-square test for association, followed by multivariable binary logistic regression analyses. Gender-specific analyses were also conducted. SPSS version 24 (IBM Corp.; Armonk, NY, USA) was used for all the statistical analyses, and an alpha less than 0.05 was used to denote significance. Cases with missing values were excluded listwise from the analyses.

## 3. Results

Table 1 displays the characteristics of the 302 study participants. A majority (63%) were women (63.0%), Hispanic (67.9%), aged 18–20 years (51.7%), worked part-time (50.7%), and were U.S.-born (76.5%). In addition, 37.5% reported food insecurity, 48.2% reported average to very poor physical health status, and another 45% reported average to very poor mental health status. Finally, 46.2% of the participants reported any psychological distress, with 21.4% reporting serious psychological distress and 24.8% reporting mild to moderate psychological distress (data not shown in table).

Table 2 displays the results of the chi-square test for the association between the presence of psychological distress and an average to very poor self-perceived mental health status for each of the independent variables. Significant differences were found in the prevalence of any psychological distress by gender, with a higher percent of any distress noted among women (51.6%), as compared to men (37.7%). Likewise, the prevalence of average to very poor self-perceived mental health status was significantly higher among women than men (52.5% vs. 34.5%).

A significant association was also found between self-perceived physical health status and the presence of psychological distress. Those with an average to very poor self-perceived physical health status had a higher prevalence of any psychological distress (55.0% vs. 37.6%). Similarly, those who reported average to poor self-perceived physical health status were also more likely to report a higher percentage of average to very poor self-perceived mental health status, as compared to participants who noted excellent/good physical health status (60.3% vs. 31.6%).

Furthermore, food insecurity and mental health outcomes were also significantly associated. When compared to their food-secure counterparts, food-insecure participants had a significantly higher prevalence of psychological distress (67.6% vs. 32.2%) and were also more likely to report an average to very poor self-reported mental health status (59.6% vs. 36.3%).

Gender-specific analyses showed no significant association between either of the negative mental health outcomes to age, ethnicity, employment status, or country of birth (data not shown), which is similar to the overall population analysis results as well. Additionally, the association between food insecurity and each of the mental health outcomes remained significant for the gender-specific analysis (data not shown).

However, unique gender-specific patterns emerged for self-perceived physical health status and alcohol use, as can be seen in Table 3.

For example, the associations between self-perceived physical health status and psychological distress and self-perceived mental health status were only significant for women. Women with average to very poor self-perceived physical health status had a higher prevalence of psychological distress (58.3% vs. 43.5%) compared to their counterparts who noted an excellent/good physical health status. Similarly, women who reported average to very poor self-perceived physical health status also were more likely to report an average to very poor self-perceived mental health status compared to their counterparts who noted an excellent/good physical health status (69.1% vs. 34.5%).

Likewise, while the overall analysis did not show an association between alcohol use and psychological distress or self-perceived mental health status, the gender-specific analysis demonstrated significant outcomes for women only in both cases. Women who reported any alcohol use in the past 12 months had a higher prevalence of psychological distress (57.3% vs. 41.0%) and a higher prevalence of reporting average to very poor self-perceived mental health status (57.5% vs. 40.4%).

Table 4 highlights the results of the multivariable binary logistic regression analyses for the overall population for each of the mental health outcomes.

Mostly consistent with bivariate analyses for the population, food insecurity, gender, and self-perceived low physical health status was associated with the presence of psychological distress among college students, even after accounting for control variables. Among those with food insecurity, both the odds of psychological distress in Table 4 (OR = 3.645, *p* < 0.05) and average to very poor self-perceived mental health status (OR = 2.687, *p* < 0.05) were higher as compared to their food-secure counterparts. As compared to men, women had higher odds of having psychological distress (OR = 2.280, *p* < 0.05) as well as reporting average to very poor self-perceived mental health status (OR = 2.700, *p* < 0.05). In addition, self-perceived low physical health status was also associated with higher odds of psychological distress (OR = 1.962, *p* < 0.05) and reporting an average to very poor self-perceived mental health status (OR = 2.807, *p* < 0.05).

A gender-specific multivariable binary logistic regression was also conducted. Table 5 displays the results for any variable that showed differences from the overall population results shown in Table 4. As such, there were no differences from Table 4 in regard to significance for age, ethnicity, employment status, and country of birth, while food insecurity remained significantly associated with negative mental health outcomes (data not shown). However, the association noted in the overall population related to physical health and alcohol was different when analyzed by gender. Among women, any alcohol use in the past 12 months was associated with reporting an average to very poor self-perceived mental health status (OR = 2.505, *p* < 0.05). Reporting a low self-perceived physical health was also associated with reporting an average to very poor mental health status among women (OR = 3.601, *p* < 0.05), but neither reached significance for men. On the other hand, among men reporting a low perceived physical health status was associated with higher odds of the presence of psychological distress (OR = 3.477, *p* < 0.05).

## 4. Discussion

Mental illness is a major public health issue, especially among young adults who share a disproportionately higher burden and low mental health service utilization (3). As such, this study aimed to assess the key factors associated with psychological distress among such a vulnerable population, especially with emphasis on the role of food insecurity, as well as any putative gender differences. The study results highlights several key findings: (i) psychological distress is substantial among college students, with gender-specific trends; (ii) alcohol use and (iii) self-perceived physical health status are both associated with some negative mental health outcomes in a gender-specific manner; and (iv) food insecurity is associated with negative mental health outcomes and is consistent across gender.

The study results show, consistent with the literature [33], that college students share a higher tax of psychological distress. For instance, in this study, 21.4% of the participants had serious psychological distress, in comparison to 3.4% of adults in the U.S. [4]. The higher odds of any distress among women in this study population as compared to men, even after accounting for control variables, also confirms existing trends among U.S. adults as noted by the CDC [4], as well as global trends [34]. Such results and the existing literature continue to emphasize the importance of mental health care among young adults, especially for women. According to WHO, gender norms may play a role in the higher rate of mental illness noted among women [35], and potential contributing factors may be power, societal position, etc.; all factors display the need for further research. For example, the data from WHO’s World Mental Health Surveys [34] note that, in recent cohorts, gender differences in major depression and related outcomes have been narrowing. As such, this has been hypothesized to be attributed to increasing gender equity and income opportunities for women, as well as more financial and health independence. Given that our study also notes this trend even among college students where education is equitably distributed, addressing gender role expectations and norms among women college students may be of value for future studies. While our study only highlights possible cross-sectional gender-specific roles, future longitudinal studies are needed to further explore gender differences. It should also be noted that the potential stigma associated with mental illness, especially among males [36], could be a contributing factor to the lower levels in men, and thus further research is needed to correctly identify the most at-risk populations. This is further evident in our results related to men’s psychological distress level, as noted below.

Among men, having a low perception of physical health status was related to presence of psychological distress and not self-perceived poor mental health status (a pattern noted among women). These unique patterns could be attributed to gender-specific patters in mental health reporting and help-seeking behavior. For example, reporting one’s own mental health status can be considered embarrassing, and previous research has shown that men are less likely to seek mental healthcare due to embarrassment [37]. As such, we hypothesize that despite feeling they have low physical health status, men were not necessarily reporting low mental health status due to the stigma associated with mental health. However, the Kessler 6 scale, which is a validated instrument in uncovering possible future serious mental illness, shed light onto the presence of distress even if such participants did not report their mental health to be poor. Thus, these men are at risk of long-term poor mental health wellbeing; early assessment through validated measures, instead of relying on help-seeking behavior, may be valuable in curbing the burden of mental illness among men.

Finally, our study results also demonstrate that food insecurity was significantly associated with the presence of psychological distress, both in the overall population and in the gender-specific analysis. Our results show that being food-insecure was associated with over 3.5 times the odds of having psychological distress, as well as over 2.5 times higher odds of perceiving one’s mental health to be average to very poor. Studies among other vulnerable populations, such as ethnic minorities, women, migrant workers, etc., have shown that food insecurity is a major social determinant of health, contributing to higher rates of obesity, poor physical health outcomes, poor nutrition, etc. [38,39,40,41], though much of the literature is focused on children. Nevertheless, our results also note that food insecurity plays a negative role in the mental health wellbeing of college students, and thus ensuring adequate and healthy access to food is warranted to provide such students necessary resources for academic success. While interventions to address food insecurity have often been providing food banks or farmers’ markets, often these are analogous to drugs—as treatment for a disease with little emphasis on prevention. Researchers have been called on to consider food insecurity as a social disease, and thus, instead of treatments, early preventive measures are needed [42]. These preventive measures may include early interventions to ensure that all at-risk students enroll in federal food assistance programs and nutritional courses on how to cook effectively on low budgets as part of college orientation, as well as, as noted in a piece by Harvard [43], having dining rooms open during break and allowing a portion of Pell Grants to aid in food, etc., may provide a scope of prevention. In addition, while the opposition of federal legislation to aid college students to become food-secure have cited the lack of nationally representative data, multi-state polices such as the proposed Closing the College Hunger Gap Act [44] would promote national data collection measures to ensure that there is an accurate snapshot of the burden of food insecurity among such a vulnerable population. The efficacy of such interventions remains to be further evaluated and highlights the need for further research on the burden of food insecurity among college students.

The results of this study should be interpreted in the context of its limitations. The student population in this study is specific to a high immigrant, first-generation, mid-size college campus and is not a representation of a national population. The data on self-perceived mental health is likely underreported due to stigma, and the true burden may be substantially higher, as noted in the difference among men. Due to the low sample size, we could not assess those who were gender non-binary, and further research is needed to address the unique barriers that college students who are gender non-binary or gender-nonconforming face in regard to mental wellbeing.

## 5. Conclusions

Notwithstanding such limitations, the results of our study not only highlight gender-specific trends in mental health wellbeing but further add to the growing body of literature on the negative impact of food insecurity on the health and wellbeing of vulnerable populations.

## Figures and Tables

**Table 1:** Characteristics of the study participants, *n* = 302.

Variable	Prevalence (%)
**Sex**	
Female	63.0
Male	37.0
**Ethnicity**	
Hispanic	67.9
Non-Hispanic	31.1
**Age**	
18–20. years	51.7
21–23 years	33.8
24 or more years	14.6
**Employment status**	
Not employed	39.6
Currently employed part-time	50.7
Currently employed full time	9.7
**Country of birth**	
U.S.-born	76.5
Foreign-born	23.5
**Past 12 month any alcohol use**	
Yes	64.6
No	35.4
**Self-perceived physical health status**	
Excellent/good	51.8
Average-very poor	48.2
Food security level	
Food-secure	62.5
Food-insecure	37.5
**Self-perceived mental health status**	
Excellent/good	55.0
Average-very poor	45.0
**Psychological distress level**	
No	53.8
Mild to moderate	24.8
Serious	21.4

**Table 2:** Factors associated with the presence of psychological distress and a low self-perceived mental health status.

Variable	Presence of Psychological Distress	Average to Very Poor Self-Perceived Mental Health Status
Sex *		
Female	51.6	52.5
Male	37.7	34.5
**Ethnicity**		
Hispanic	45.5	44.8
Non-Hispanic	47.8	46.7
**Age**		
18–20 years	45.0	47.3
21–23 years	49.0	47.5
24 or more years	44.2	34.1
**Employment status**		
Not employed	46.9	46.9
Currently employed part-time	44.5	47.0
Currently employed full time	51.9	33.3
**Country of birth**		
U.S.-born	45.2	44.7
Foreign-born	50.0	45.6
**Past 12 month any alcohol use**		
Yes	48.9	48.7
No	40.0	36.7
Self-perceived physical health status *		
Excellent/good	37.6	31.6
Average-very poor	55.0	60.3
Food security level *		
Food-secure	32.2	36.3
Food-insecure	67.6	59.6

* <0.05.

**Table 3:** Factors ^a^ associated with the presence of psychological distress and low self-perceived mental health status, by sex.

Variable	Presence of Psychological Distress		Average to Very Poor Self-Perceived Mental Health Status	
	Female	Male	Female	Male
Past 12 months any alcohol use *				
Yes	57.3%	34.9%	57.5%	33.8%
No	41.0%	38.5%	40.4%	31.7%
Self-perceived physical health status *				
Excellent/good	43.5%	30.2%	34.5%	28.1%
Average-very poor	58.3%	48.8%	69.1%	43.5%

^a^ Factors are displayed if associations were different from the overall population analysis (Table 2); * Significant for females only.

**Table 4:** Odds ratio of psychological distress and low self-perceived mental health status.

Variable	Presence of Psychological Distress		Average to Very Poor Self-Perceived Mental Health Status	
	Odds Ratio	*p*-Value	Odds Ratio	*p*-Value
**Sex**				
Female	2.280	0.006	2.700	0.001
Male	Reference		Reference	
**Ethnicity**				
Hispanic	0.808	0.503	0.681	0.226
Non-Hispanic	Reference		Reference	
**Age**				
18–20 years	1.395	0.448	2.136	0.091
21–23 years	1.151	0.754	1.544	0.342
24 or more years	Reference		Reference	
**Employment status**				
Not employed	Reference		Reference	
Currently employed part-time	0.796	0.460	1.029	0.925
Currently employed full-time	0.820	0.713	0.371	0.085
**Country of birth**				
U.S.-born	Reference		Reference	
Foreign-born	1.672	0.134	1.397	0.326
**Past 12 month any alcohol use**				
Yes	1.625	0.128	1.820	0.056
No	Reference		Reference	
**Self-perceived physical health status**				
Excellent/good	Reference		Reference	
Average-very poor	1.962	0.017	2.807	<0.001
**Food security level**				
Food-secure	Reference		Reference	
Food-insecure	3.645	0.000	2.687	0.001

**Table 5:** Odds ratio of factors ^a^ associated with the presence of psychological distress and low self-perceived mental health status, by sex.

Variable	Presence of Psychological Distress		Average to Very Poor Self-Perceived Mental Health Status	
	Female	Male	Female	Male
**Past 12 month any alcohol use**				
Yes	OR = 2.262 *p* = 0.050	OR = 0.964 *p* = 0.948	OR = 2.505 *p* = 0.029	OR = 1.300 *p* = 0.600
No	Reference	Reference	Reference	Reference
**Self-perceived physical health status**				
Excellent/good	Reference	Reference	Reference	Reference
Average-very poor	OR = 1.636 *p* = 0.150	OR = 3.477 *p* = 0.034	OR = 3.601 *p* < 0.001	OR = 2.003 *p* = 0.175

^a^ Factors are displayed if the significance of association was different from the overall population analysis (Table 4).
